# The views of teenagers with obesity, their caregivers, and doctors: a plain language summary of the ACTION Teens global survey

**DOI:** 10.2217/cer-2022-0164

**Published:** 2022-10-20

**Authors:** Vicki Mooney, Louise A Baur, Abdullah Bereket, Bassam Bin-Abbas, Walter Chen, Fernando Fernández-Aranda, Nayely Garibay Nieto, Juan Pedro López Siguero, Claudio Maffeis, Cynthia Karenina Osorto, Ricardo Reynoso, Young-Jun Rhie, Martín Toro-Ramos, Jason CG Halford

**Affiliations:** 1 The European Coalition for People Living with Obesity (EASO ECPO), Dublin, Ireland; 2 Patient author; 3 Children's Hospital Westmead Clinical School, The University of Sydney, Westmead, New South Wales, Australia; 4 Department of Pediatrics, Division of Pediatric Endocrinology & Diabetes, Marmara University School of Medicine, Istanbul, Turkey; 5 Department of Paediatrics, King Faisal Specialist Hospital & Research Center, Riyadh, Saudi Arabia; 6 Children's Hospital, China Medical University, Taichung, Taiwan; 7 Department of Psychiatry, University Hospital of Bellvitge-IDIBELL & CIBEROBN, Barcelona, Spain; 8 Pediatric Obesity Clinic & Wellness Unit, Hospital General de México “Dr. Eduardo Liceaga”, Mexico City, Mexico, & Hospital Angeles del Pedregal, Mexico City, Mexico; 9 Pediatric Endocrinology Unit, Regional University Hospital, Málaga, Spain; 10 Department of Surgery, Dentistry, Gynecology, & Pediatrics, Section of Pediatric Diabetes & Metabolism, University of Verona, Verona, Italy; 11 Global Medical Affairs, Novo Nordisk A/S, Copenhagen, Denmark; 12 Medical & Scientific Affairs, International Operations, Novo Nordisk Health Care AG, Zürich, Switzerland; 13 Department of Pediatrics, Korea University College of Medicine, Seoul, South Korea; 14 Outpatient Department, Hospital Alma Mater de Antioquia, Medellin, Colombia; 15 School of Psychology, University of Leeds, Leeds, UK

**Keywords:** adolescents, caregivers, doctors, healthcare professionals, lay summary, obesity, plain language summary, survey, teenagers, weight management, weight

## Abstract

**What is this summary about?:**

This is a summary of a research survey called ACTION Teens. In our survey, 12,987 people from 10 countries answered questions about obesity. They were: 5275 teenagers with obesity, 5389 caregivers of teenagers with obesity, and 2323 doctors who provide medical care for teenagers with obesity.

**What were the main results of the survey?:**

Most teenagers with obesity were worried about their weight and thought that losing weight was their responsibility. Many teenagers had already tried to lose weight. For teenagers, wanting to be more fit or in better shape was the top reason for wanting to lose weight. Some caregivers did not realize how worried their teenager was about their own weight. There were also some caregivers who were not aware of their teenager's recent attempts to lose weight. As a group, the doctors did not know the main reasons why teenagers want to lose weight. They also did not know the main reasons preventing teenagers from losing weight.

**What do the results of the survey mean?:**

Teenagers with obesity will be better supported and understood if there is better communication between teenagers, caregivers, and doctors.

**Clinical Trial Registration**: NCT05013359 (ClinicalTrials.gov)

To read the full Plain Language Summary of this article, click here to view the PDF.

Link to original article here

